# Evaluation of a Subject specific dual-transmit approach for improving B_1_ field homogeneity in cardiovascular magnetic resonance at 3T

**DOI:** 10.1186/1532-429X-15-68

**Published:** 2013-08-06

**Authors:** Ramkumar Krishnamurthy, Amol Pednekar, Marc Kouwenhoven, Benjamin Cheong, Raja Muthupillai

**Affiliations:** 1 Department of Bioengineering, Rice University, Houston, TX, USA; 2 Philips Healthcare, Houston, TX, USA; 3 Philips Healthcare, Best, The Netherlands; 4 Department of Radiology, Texas Heart Institute at St. Luke's Episcopal Hospital, 6720 Bertner Avenue, MC 2-270, Houston, TX 77030, USA

**Keywords:** Cardiovascular magnetic resonance, Dual-transmit radiofrequency system, B_1_ field shimming, Patient body habitus

## Abstract

**Background:**

Radiofrequency (RF) shading artifacts degrade image quality while performing cardiovascular magnetic resonance (CMR) at higher field strengths. In this article, we sought to evaluate the effect of local RF (B_1_ field) shimming by using a dual-source–transmit RF system for cardiac cine imaging and to systematically evaluate the effect of subject body type on the B_1_ field with and without local RF shimming.

**Methods:**

We obtained cardiac images from 37 subjects (including 11 patients) by using dual-transmit 3T CMR. B_1_ maps with and without subject-specific local RF shimming (exploiting the independent control of transmit amplitude and phase of the 2 RF transmitters) were obtained. Metrics quantifying B_1_ field homogeneity were calculated and compared with subject body habitus.

**Results:**

Local RF shimming across the region encompassed by the heart increased the mean flip angle (μ) in that area (88.5 ± 15.2% vs. 81.2 ± 13.3%; P = 0.0014), reduced the B_1_ field variation by 42.2 ± 13%, and significantly improved the percentage of voxels closer to μ (39% and 82% more voxels were closer to ± 10% and ± 5% of μ, respectively) when compared with no RF shimming. B_1_ homogeneity was independent of subject body type (body surface area [BSA], body mass index [BMI] or anterior-posterior/right-left patient width ratio [AP/RL]). Subject specific RF (B_1_) shimming with a dual-transmit system improved local RF homogeneity across all body types.

**Conclusion:**

With or without RF shimming, cardiac B1 field homogeneity does not depend on body type, as characterized by BMI, BSA, and AP/RL. For all body types studied, cardiac B_1_ field homogeneity was significantly improved by performing local RF shimming with 2 independent RF-transmit channels. This finding indicates the need for subject-specific RF shimming.

## Background

Despite the increasing availability of 3T systems in the clinical setting, 1.5T remains the field-strength of choice for routine clinical cardiovascular magnetic resonance (CMR). Cardiac imaging at 3T is hampered by more pronounced off-resonance related artifacts in commonly used CMR sequences such as balanced steady state free precession (b-SSFP). It is also hampered by signal non-uniformity across the imaging slice for radiofrequency (RF) intensive sequences such as black-blood turbo-spin echo (BB-TSE), due to transmit RF field (B_1_) inhomogeneity. A number of methods have been proposed in the literature to combat these effects [1-8]. A particularly important problem is the transmit B_1_ field variation across the slice at 3T in body and cardiac imaging, as the wavelength of the RF field at 3T approaches the size of the human body. Sung and coworkers demonstrated that the transmit B_1_ field can vary by as much as 50% across the heart at 3T, and the resulting loss of contrast is irreversible [4]. Various investigators have attempted to relate the extent of such RF inhomogeneity to the patient’s body habitus. Some have also proposed the use of RF cushions to minimize RF shading in body and cardiac imaging at 3T [1,9,10]. Over the years, several groups have proposed that RF homogeneity can be improved by using independently controlled multiple-transmit sources and have demonstrated the feasibility of this approach in both phantom and human studies [11,12]. Recently, Willinek and associates [12] showed that using a dual-transmit approach in a clinical scanner markedly improved the quality of body images. To our knowledge, however, the literature contains only 1 previous article regarding the use of dual-transmit systems for cardiac imaging at 3T [13].

The purposes of our current study were 1) to observe the effect of local RF shimming for cardiac imaging using a dual-source transmit RF system that offers independent control of the RF phase and amplitude of the 2 channels, 2) to study the effect of subject body type on the B_1_ field with and without local RF shimming, and 3) to investigate the effect of local RF shimming on B_1_ field inhomogeneity in cardiac cine imaging using b-SSFP sequence.

## Methods

### Subjects

Thirty-seven subjects (26 asymptomatic/healthy volunteers and 11 patients) underwent CMR in this prospective study. The subjects included 27 men and 10 women, with a mean age of 46.2 ± 17.3 years (range, 17–76 years). The study was approved by our hospital’s (St. Luke’s Episcopal Hospital) institutional review board and complied with the requirements of the Health Insurance Portability and Accountability Act. All subjects provided written informed consent on enrolling in the study. No subject data obtained were excluded. We measured the height and weight of each subject, as well as the anterior-posterior (AP) and right-left (RL) dimensions, which were obtained from axial images that included both ventricles. The AP and RL measurements were made with the subject lying supine in the scanner. From these measurements, the following characteristics pertaining to body type were calculated for each subject: a) body surface area (BSA), expressed as [m^2^] using the Mosteller formula [14]: BSA = (weight(kg) * height(cm)/3600)^(0.5)^; b) body mass index (BMI) expressed as [kg/m^2^], and c) anterior-posterior/right-left (AP/RL) ratio, from the measured AP and RL dimensions.

### Image acquisition

All imaging took place on a 70-cm bore 3T MR scanner (Ingenia, Philips Healthcare, Best, The Netherlands) which has a 2-channel transmit system with independent RF control. All data acquisition was vector-cardiography (VCG) gated. For signal reception, we used a combination of 12 channels from the table-top integrated digital posterior coil, up to 4 additional posterior channels from the head-neck base coil, and 16 channels from the digital anterior coil. After obtaining the initial scout views, we acquired cine images of the heart in the long-axis, 4-chamber, and short-axis views.

### B_1_ map acquisition

B_1_ maps of the axial plane across the heart were generated based on a cardiac gated saturation recovery prepared dual flip angle method described previously [15,16]. The dual flip angle method was used in combination with echo planar imaging (EPI) readout and sensitivity encoding SENSE acceleration. Other authors [4] also have used the dual flip angle method to quantify the cardiac B_1_ field. The acquisition parameters were: repetition time (TR)/echo time (TE) = 1000/5.7 ms; target flip angles = 60°/120°; acquired voxel size = 5*10*10 mm^3^; FOV=520×700 mm; and EPI readout factor = 11; SENSE (acceleration factor of 2) for parallel image acquisition; Spectrally Attenuated Inversion Recovery (SPAIR) pulse for fat suppression; and a WET (Water suppression Enhanced through T_1_ effects – multiple RF pulses followed by dephasing gradients to perform effective saturation over a range of T_1_ species and B_1_inhomogeneities) saturation pre-pulse delay of around 500 ms depending on the subject’s heart rate. Prospective cardiac triggering was used. Scan time for the B_1_ maps was 7–9 seconds (depending on the heart rate). B_1_ calibration scans consisted of 2 sequential B_1_ map acquisitions combined in a single scan, each with only one of the RF transmission channels switched on, in order to determine the B_1_ maps per transmission channel. After acquisition of the calibration scan, the complex B_1_ data was used to set the amplitude and phase settings of the 2 independent transmit channels. The optimal RF shim setting for each subsequent scan was determined automatically by the vendor-provided MR scanner software. This software uses a minimal cost algorithm to minimize the coefficient of signal variation of the B_1_ field based on the linear combination of phase and amplitude of the different RF transmit channels, based on the B_1_ data from the calibration scan within the user-defined local shim area. B_1_ map with RF shimming was acquired with the optimized RF shim settings. For comparison, another B_1_ map without RF shimming was also acquired by operating the RF transmit system in conventional quadrature mode, in which the 2 transmit sources had a fixed phase difference of 90° and had identical transmit power.

### Cine b-SSFP images of the heart

Cine b-SSFP images were acquired with local RF shimming near the base of the left ventricle in the short-axis orientation for all the subjects. For 12 of the 37 subjects (chosen randomly), comparative cine b-SSFP images without RF shimming were also acquired at an identical location. The acquisition parameters were: TR/TE = 2.6/1.3 ms; α = 45°; turbo factor = 15; field of view (FOV) = 320*320 mm^2^; Acquired voxel size ~ 2*1.7*8 mm^3^; cardiac phases per cycle ~25.

### Data analysis

An author of this study with more than 3 years’ experience in cardiac CMR imaging performed the data analysis.

### Quantitative evaluation of B_1_ maps

**Figure 1:**
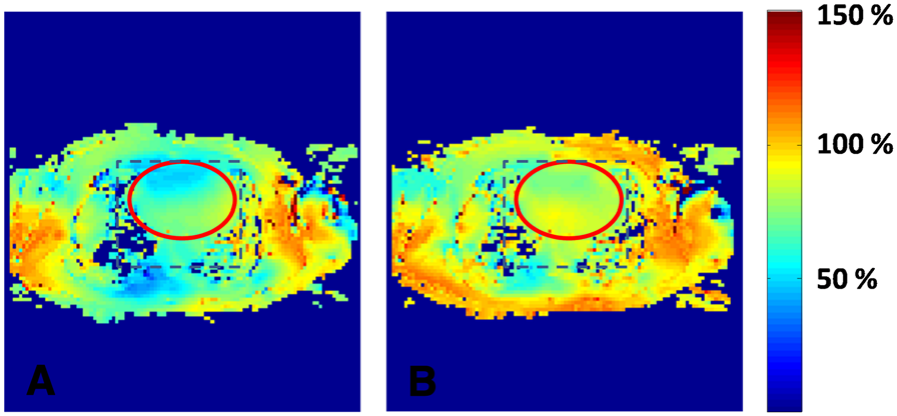
**B**_**1**_**maps of an axial slice across the heart without (A) and with (B) local RF shimming.** The values are expressed as percentage of the intended flip angle experienced. The rectangular box corresponds to the region being shimmed. An elliptical region of interest (ROI) was manually drawn circumscribing the heart. Metrics characterizing B_1_ field homogeneity were calculated within this ROI.

On the B_1_ maps generated (as a percentage of intended flip angle) with and without local RF shimming, the region-of-interest (ROI) circumscribing the heart (Figure 1) was manually drawn using a custom-built software program (MATLAB™; The Mathworks, Natick, Massachusetts). The following metrics were used for quantitative evaluation of RF homogeneity: 1) The mean of the percentage of the intended flip angle within the ROI (μ); 2) the coefficient of signal variation (C_v_), defined as the ratio of the standard deviation (σ) to the mean (μ) of the voxels within the ROI, where a lower ratio indicates more uniformity; and 3) a cumulative histogram that shows the fraction of the total number of voxels that fall within a specific value of the mean (expressed as a percentage); a higher count at a given threshold corresponds to a more uniform B_1_ field distribution. Also, the variation of μ and C_v_ with metrics that characterized the subject’s body type was studied.

### Dependence on subject body type

The transmit amplitudes and the phase of the 2 independent RF sources were tabulated for all the subjects when the B_1_ maps were acquired. The transmit amplitudes were expressed as the power amplitude ratio (20 * log (A_1_/ A_2_)), while the phase difference between the 2 RF sources was expressed as a relative phase, where the pure quadrature mode (90° phase shift) was used as the reference. In other words, if the actual phase difference between the 2 RF transmitters was 90°, the relative phase is 0°. The power amplitude ratio and relative phase values were plotted against each other to determine the spread of their variation across subject body types.

### Effect on b-SSFP cine images

**Figure 2:**
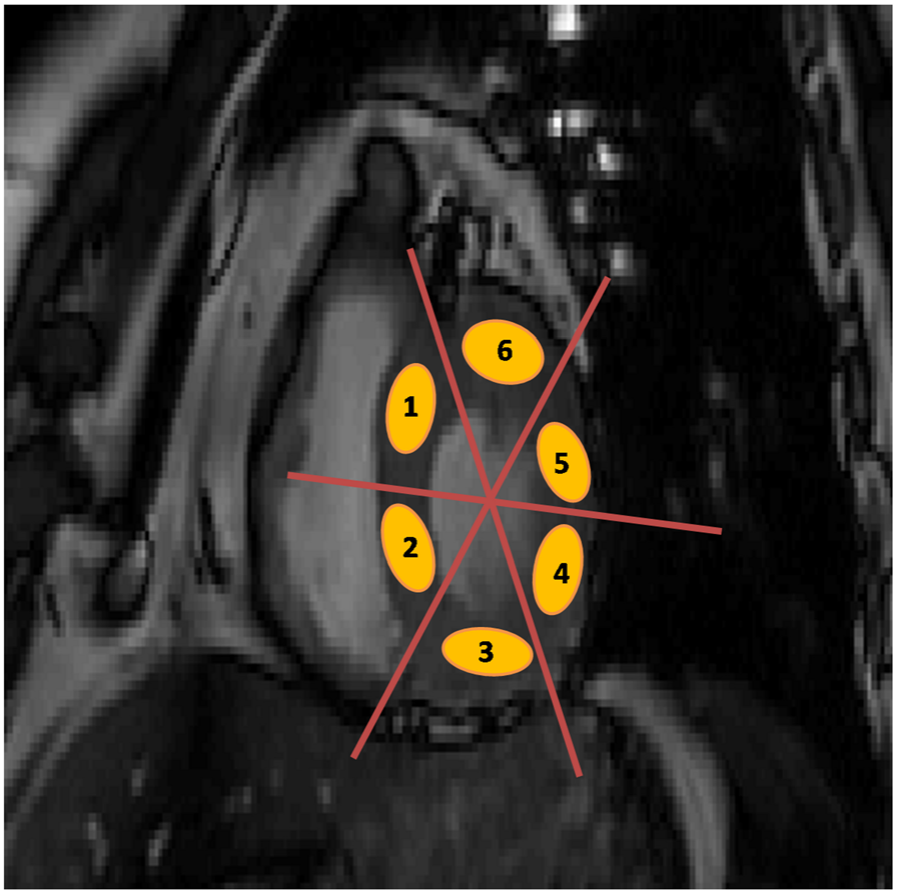
**Location of six manually drawn regions of interest (ROIs) on the let ventricle.** The myocardium in the short-axis view of the near-basal location in b-SSFP cine acquisition is shown. The measurements were made in systole.

In the b-SSFP images, 6 ROIs within the left ventricular myocardium (Figure 2) were drawn by using custom-built software (MATLAB). The regional mean intensity values and standard deviation (SD) were calculated for each patient. From these values, a percentage integral image uniformity index (IU) for each patient was calculated as: 100 * (S_max_ – S_min_)/ (S_max_ + S_min_) %; where, S_max_ is the maximum signal intensity of the 6 measured ROIs, and S_min_ the minimum signal intensity. A lower IU value corresponds to a more uniform image. The IU values obtained with and without RF shimming were compared with each other.

## Results

### Study population

The body habitus of the subjects spanned a range from normal weight to obese, as reflected by BSA (mean ± SD, 2.04 ± 0.26; range, 1.64–2.67), BMI (mean ± SD, 28.8 ± 7.2; range, 21.4–52.9), and AP/RL ratio (mean ± SD, 0.64 ± 0.06; range, 0.5–0.78).

### Quantitative evaluation of B_1_ maps

**Figure 3:**
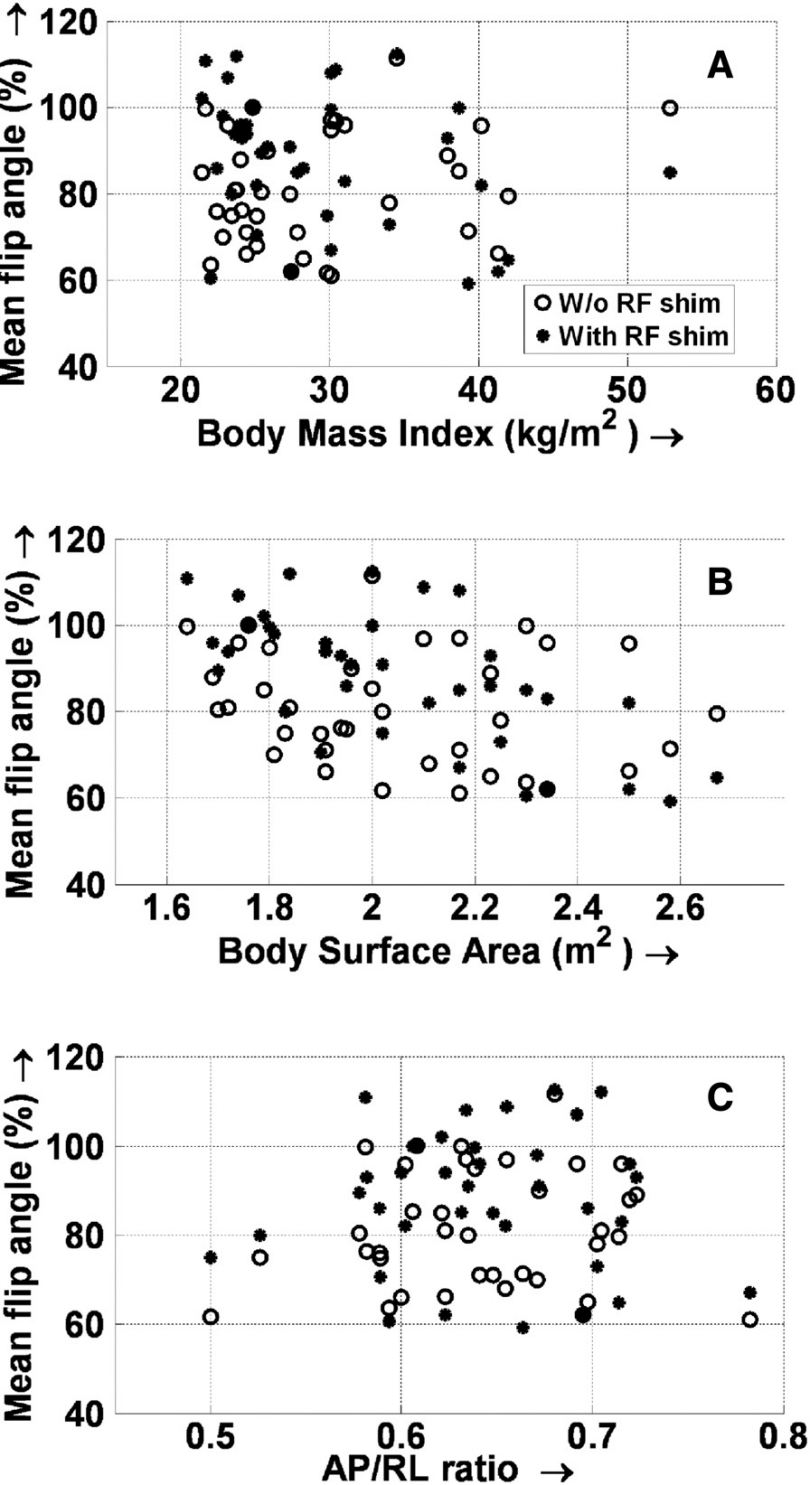
**Variation of the mean percentage flip angle with subject body habitus.** The variation of μ with body mass index **(A)**, body surface area **(B)**, and anterior-posterior/right-left (AP/RL) ratio **(C)** is shown. Because no clear pattern of variation can be discerned, the mean flip angle does not appear to depend on body type. RF, radiofrequency; W/o, without.

**Figure 4:**
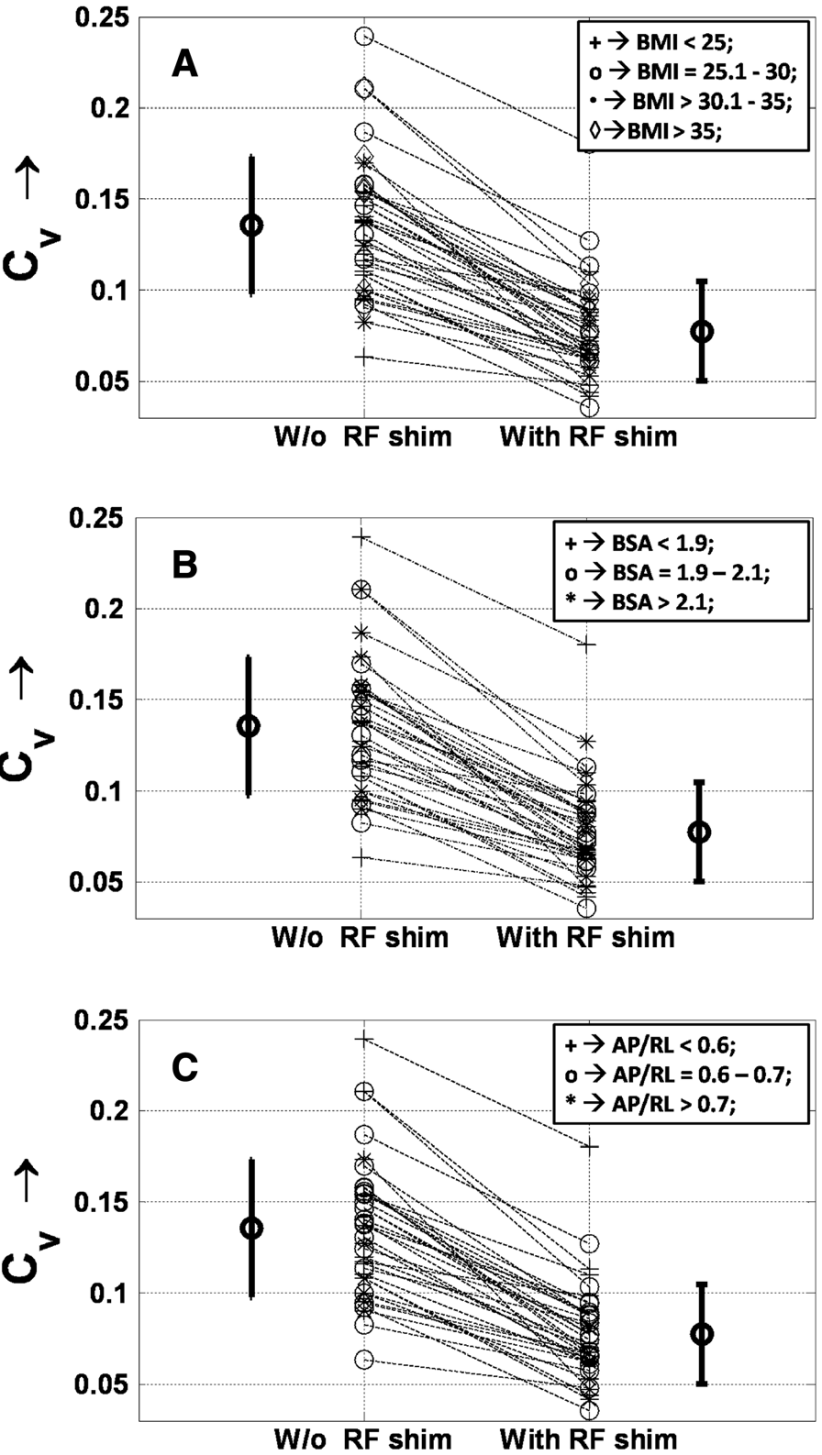
**Nomogram plotting the coefficient of signal variation (C**_**v**_**) across the region of interest for all body types.** An average decrease of 42 ± 13% is seen (*P* < 0.000001; paired Student *t* test), corresponding to a more uniform B_1_ field in case of subject-specific radiofrequency (RF) shimming. Changes in C_v_ with body mass index (BMI) **(A)**, body surface area (BSA) **(B)**, and anterior-posterior/right-left ratio (AP/LR) **(C)** are shown. As with the mean flip angle, a reduction in C_v_ is not dependent on body type. The number of subjects with 1a) *BMI < 25 kg/m*^*2*^:**14**; 1b) *BMI = 25.1–30 kg/m*^*2*^:**9**; 1c) *BMI =30.1–35 kg/m*^*2*^*:***7**; 1d) *BMI > 35 kg/m*^*2*^:**7**; 2a) *BSA < 1.9 m*^*2*^: **11**; 2b) *BSA = 1.9–2.1 m*^*2*^: **11**; 2c) *BSA> 2.1 m*^*2*^*:***15**; 3a) *AP/RL < 0.6*:**8**; 3b) *AP/RL = 0.6–0.7*:**22** ; 3c) *AP/RL >0.7:***7**. W/o, without.

**Figure 5:**
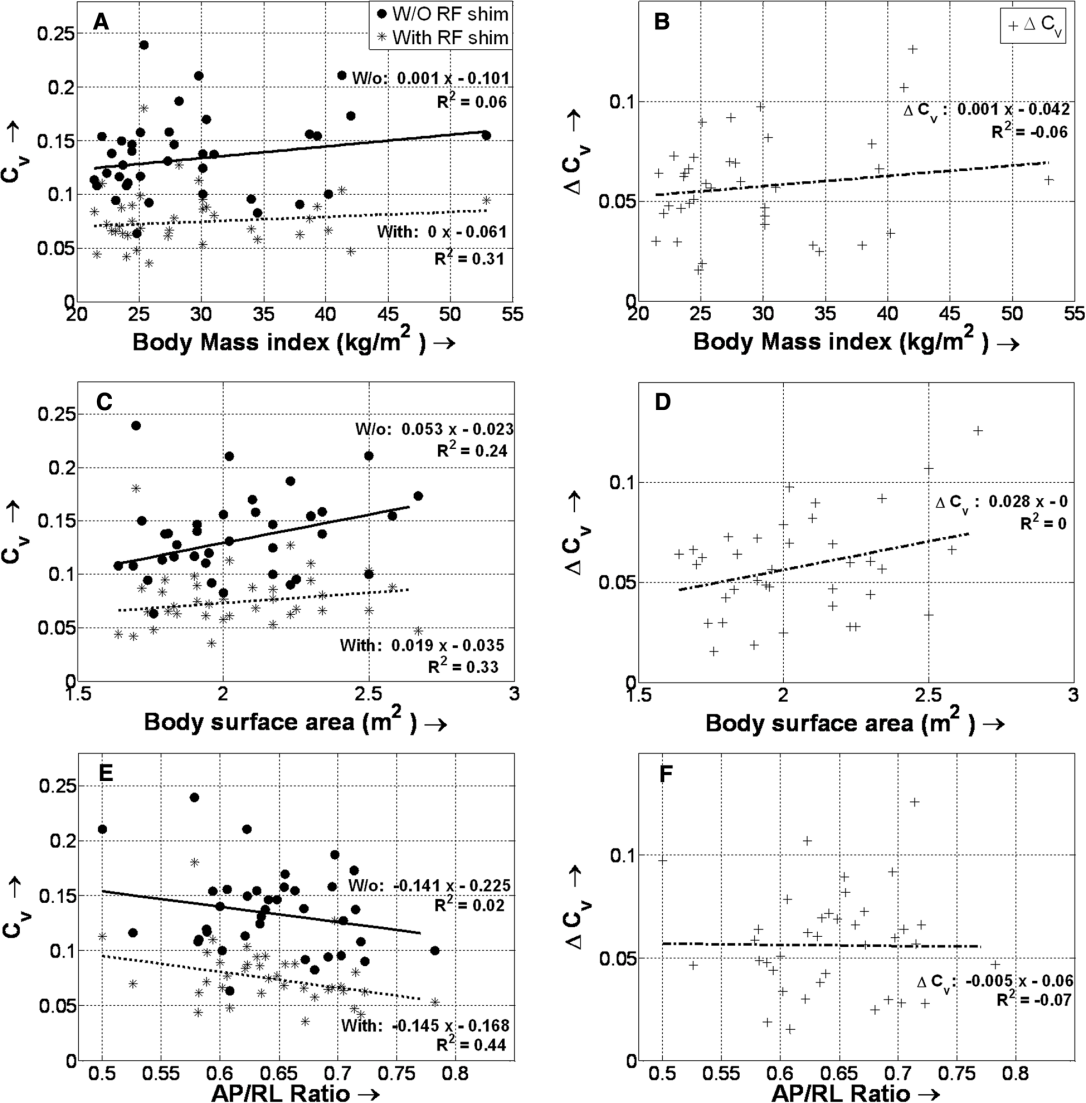
**Changes in the coefficient of signal variation (C**_**v**_**) with and without (W/o) radiofrequency (RF) shimming for different body types.** Also shown is the variation of the differences in C_v_ values (ΔC_v_) for different body types – varying body mass indexes **(A**, **B)**, body surface areas **(C**, **D)**, and anterior-posterior/right-left ratios (AP/RL) **(E**, **F)** are shown. No significant relationship is found between C_v_ (and ΔC_v_) and body type, either with or without local RF shimming.

**Figure 6:**
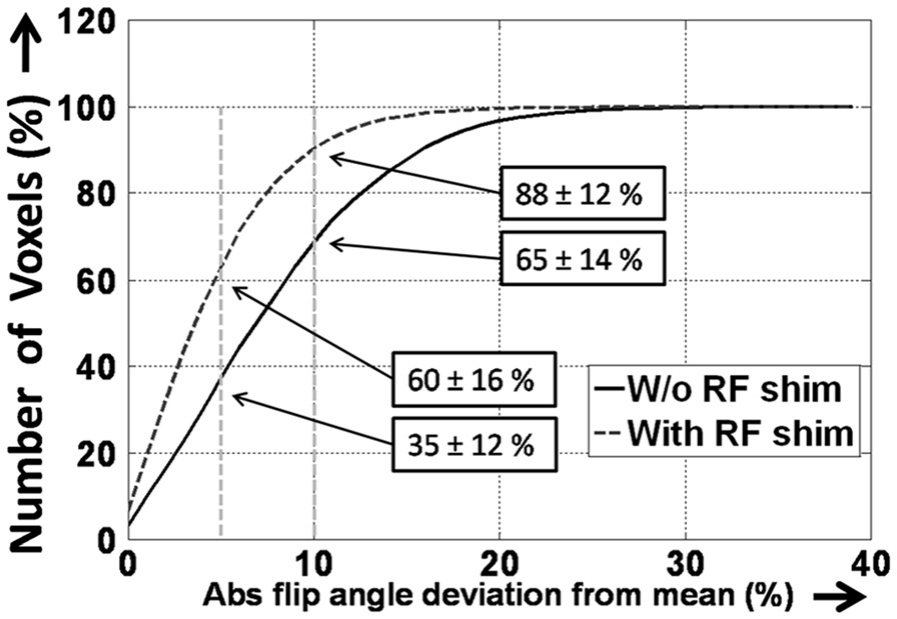
**Cumulative histogram plottingthe total number of voxels that fall within a given range from the mean flip angle in the selected region of interest (ROI).** The dotted vertical lines indicate the ± 5% and ± 10% limits. In the case of subject-specific local radiofrequency (RF) shimming, 88 ± 12% of the voxels inside the ROI fall within ± 10% of the mean value. This value decreases significantly when no RF shim is used. Abs, absolute; W/o, without.

1. The mean flip angle (μ, expressed as a percentage of the intended flip angle) across the heart increased from an average value of 81.2 ± 13.3% to 88.5 ± 15.2% when local RF shimming was used, indicating that more voxels within the ROI had a flip angle closer to the intended flip angle. This difference was significant (*P* = 0.0014; paired Student *t* test).

2. The data were analyzed to see whether there was any observable dependence between the mean flip angle and the subject body type. No particular relationship was observed between these variables, either with or without local RF shimming (Figure 3). Also, analysis of the mean flip angle and the height and weight of all the subjects did not reveal any significant interrelationship among these variables (not shown).

3. The C_v_ values revealed better RF homogeneity in each subject with subject-specific local RF shimming (Figures 4 and 5). The average C_v_ for the 37 subjects improved by 42.2 ± 13% with RF shimming (from 0.13 ± 0.03 to 0.07 ± 0.02; *P* < 0.0001; paired Student *t* test). Figure 5 shows the variation of C_v_, both with and without local RF shimming, and the change in Cv (between with and without local RF shimming) with body type. No relationship could be found between these factors. Like the mean flip angle, C_v_ values were also compared to the height and weight of the subjects individually, and no significant interrelationship could be found (not shown in Figure 5).

4. With local RF shimming, 88 ± 12% of the voxels within the ROI fallwithin ± 10% of the mean flip angle, and 60 ± 16% of the voxels fallwithin ± 5% of that angle (Figure 6). In comparison, without RF shimming, the respective values were 65 ± 14% and 35 ± 12%. This resulted in an increase of 39 ± 28% in the number of voxels that fell within ± 10% of the mean flip angle and an increase of 82 ± 66% in the number of voxels that fell within ± 5% of that angle. This difference is significant (*P* < 0.0001 in both cases; paired Student *t* test).

### Dependence on subject body type

**Figure 7:**
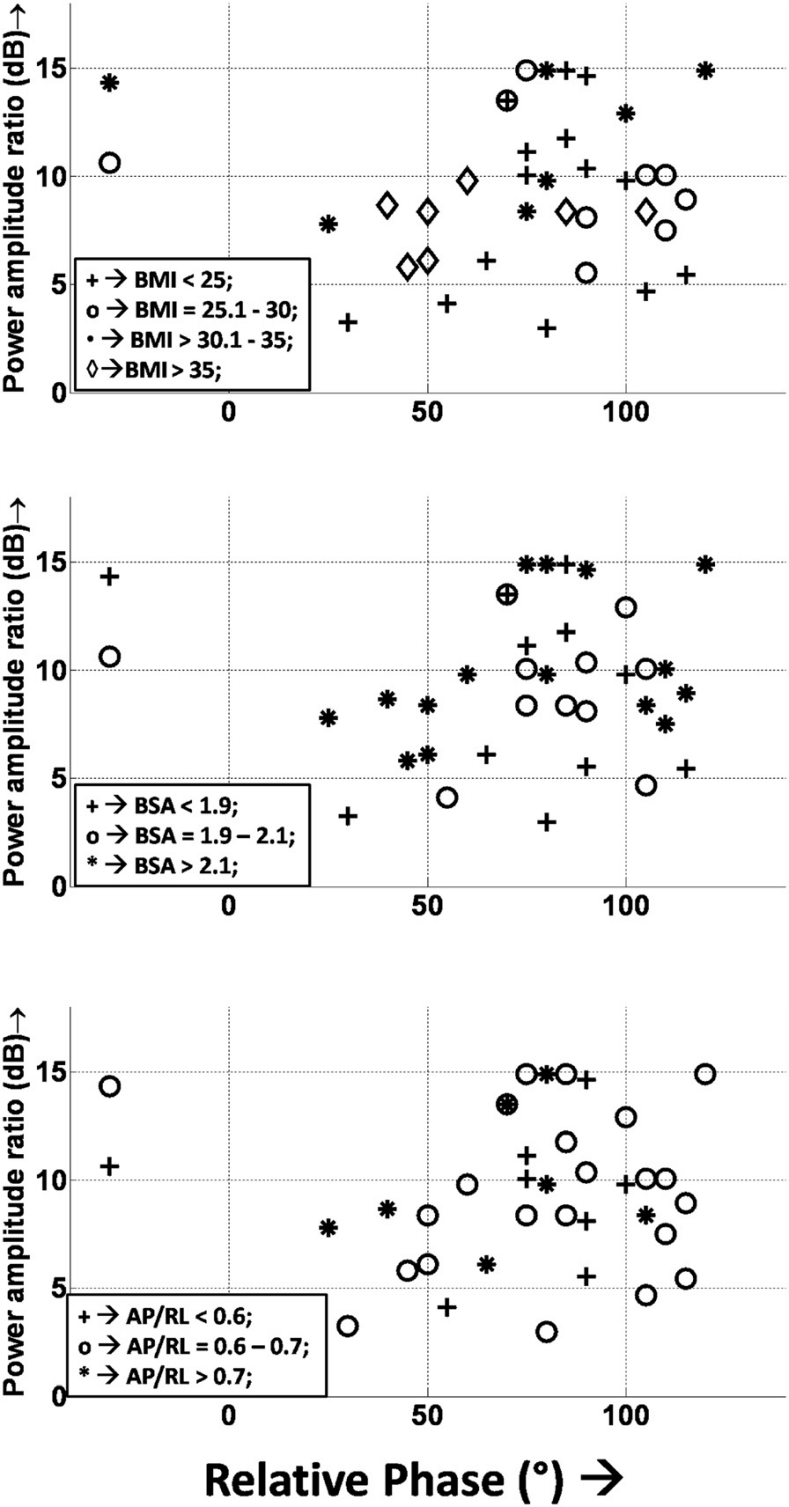
**Plot showing the power amplitude ratio (expressed in dB) with variation of the relative transmit phase in all 37 subjects.** A wide variation is seen in both the relative phase and amplitude in all subjects. Changes with body mass index (BMI) **(A)**, body surface area (BSA) **(B)**, and anterior-posterior/right-left ratio (AP/LR) **(C)** are shown. No clear pattern of variation is found between the spread of amplitude/phase and subject body type.

The transmit power amplitude ratio plotted against the relative phase showed a significant spread in the values for different subjects (Figure 7). The amplitude power ratio varied from 2.97 to 14.89 dB, while the relative phase variation between the 2 RF sources ranged from −30° to +120°. Variation of the transmit power amplitude ratio with the relative phase did not depend on the subject body type (Figure 7).

### Effect on b-SSFP cine images

**Figure 8:**
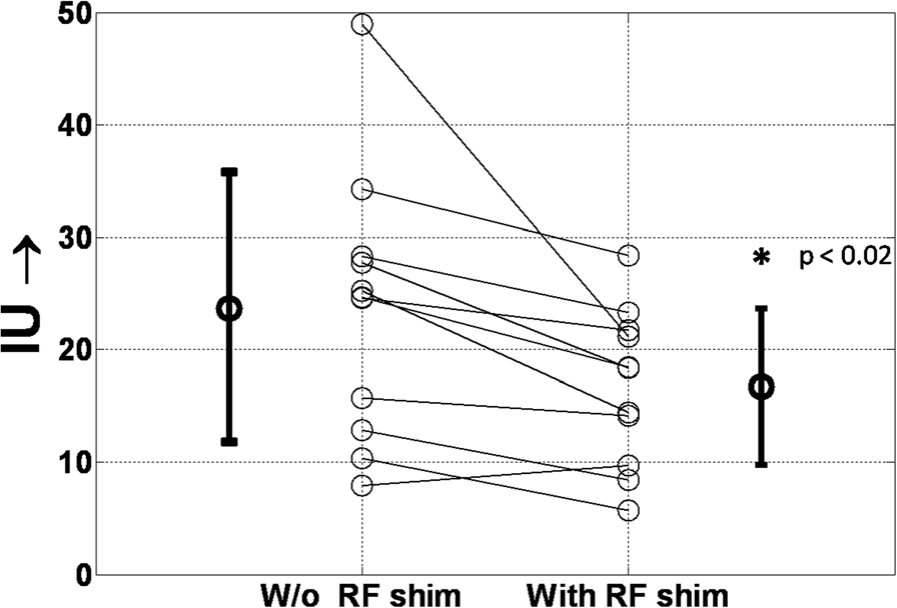
**The Image Integral Uniformity Values.** The Image Integral Uniformity (IU) was calculated for each patient image obtained with and without local RF shimming. A significant reduction in inhomogeneity is noticed when local RF shimming is employed (23.7 ± 11.8 vs. 16.7 ± 6.9). This reduction is statistically significant (p = 0.013; paired Student’s t-test) and highlights the beneficial effects of local RF shimming on image signal homogeneity.

**Figure 9:**
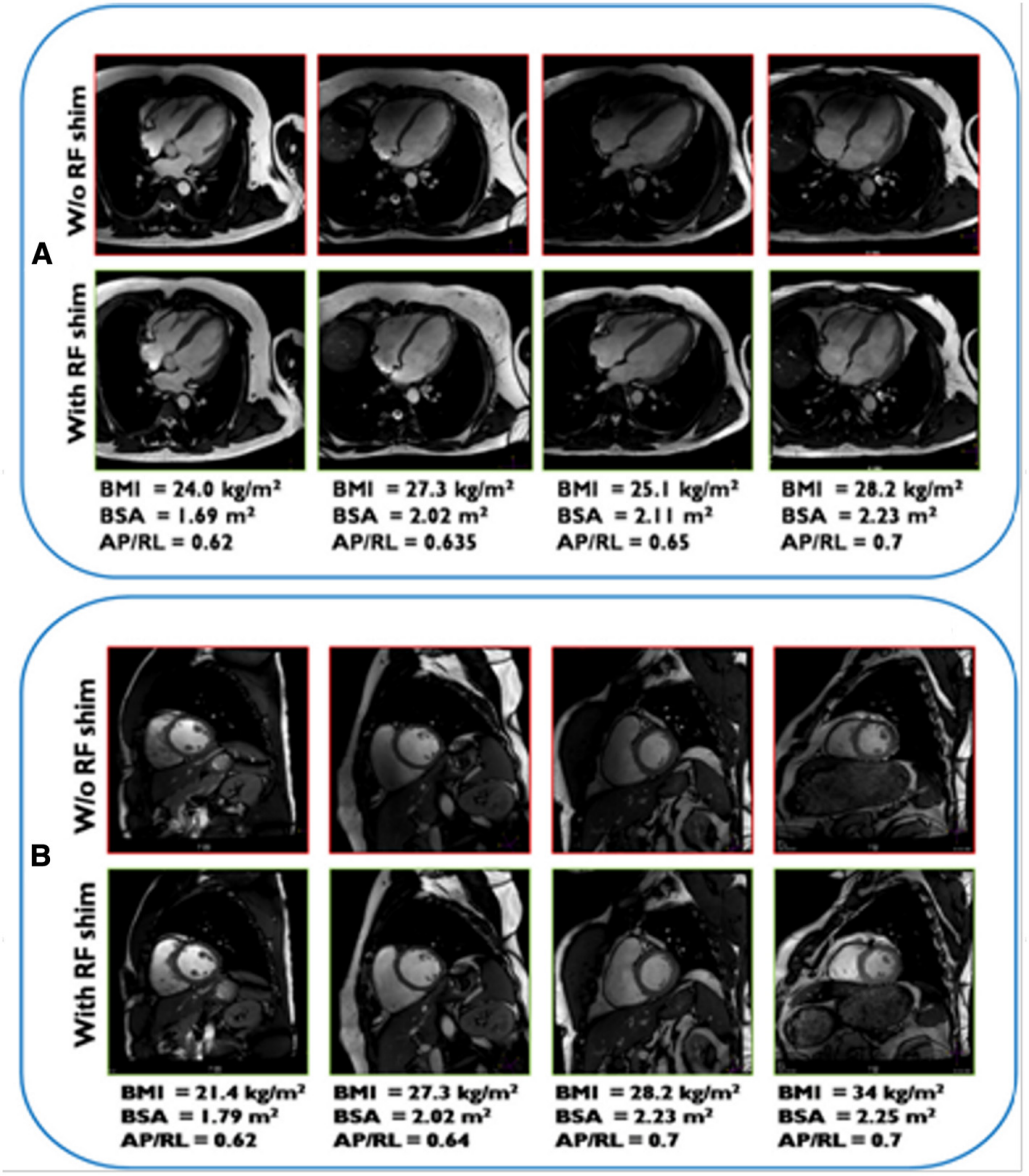
**Representative balanced steady-state free-precession images of the left ventricle.** The 4-chamber **(A)** and short-axis **(B)** views from subjects with various body types, shown in increasing order of BSA (Left to Right) is shown. Without (W/o) radiofrequency (RF) shimming, B_1_ field inhomogeneity is observed (top row) in both **A** and **B**; this inhomogeneity is reduced with RF shimming (bottom row). BMI, body mass index; BSA, body surface area; AP/RL, anterior-posterior/right-left ratio.

The IU values showed that images obtained without local RF shimming had a higher inhomogeneity of signal when compared to those obtained with local RF shimming (23.7 ± 11.8 vs 16.7 ± 6.9), and were statistically significantly different (p < 0.02, paired Student’s T-test) (See Figure 8). Figure 9 shows representative images that confirm the improvements due to local RF shimming using a dual-transmit system. Note the substantial shading artifact seen near the anterior chest wall and right ventricle without local RF shimming.

## Discussion

High-field imaging (at 3T) has gained widespread acceptance and is routinely used in higher-spatial-resolution brain imaging, neuroimaging (fMRI) [17], and musculo-skeletal imaging [18]. While the increased signal-to-noise ratio and contrast-to-noise ratio available with 3T could benefit signal-starved CMR acquisitions such as myocardial perfusion imaging, viability (late enhancement) imaging, and high-resolution coronary artery imaging, clinical acceptance of CMR at 3T has been challenging due to the presence of more-prominent artifacts in commonly used sequences such as b-SSFP (for cine imaging) and TSE (black-blood imaging). Whereas the need for greater control over B_1_ high-field imaging has been well recognized for more than a decade, clinical 3T scanners with dual-transmit ability have been available on the market only since 2009. Commercial scanners that can perform B_1_ shimming over the heart (cardiac gated B_1_ shimming) became available only in 2011. Therefore, until recently, the preferred field strength for CMR was not 3T [19]. In particular, many nonacademic radiologists remain strongly convinced that 3T is not suitable for clinical cardiovascular imaging, based on initial clinical experience with the first generation of 3T systems.

In this study, we sought to quantitatively evaluate the effect of local RF shimming using a commercially available dual-transmit system, and the effect of the subject body habitus on such shimming. Recently, Mueller and associates [13] also evaluated the effect of a dual-transmit system on the cardiac B_1_ field on 13 subjects, and their results also broadly confirm the findings of this study. To our knowledge, our current study is the first to systematically evaluate the effect local RF shimming using a dual-transmit system on subjects with varying body habitus, and to quantitatively measure the extent of the variation in B_1_ amplitude/phase necessary to effect local RF shimming in a clinical system. Several important findings of our study merit discussion.

First, cine b-SSFP sequences pose a particular challenge for CMR at 3T. For a given degree of magnetic field inhomogeneity, for the cine b-SSFP sequence to have the same extent of off-resonance–induced effects as at 1.5T, the repetition time (TR) of the sequence has to be reduced by a factor of 2. The typical TR for a b-SSFP sequence at 1.5T is around 3.0ms. Reducing the already short TR by a factor of 2 places a severe burden on the gradient hardware and imposes a significant specific-absorption-rate (SAR) constraint by increasing the RF duty cycle. Conventional approaches for lowering the SAR by using longer RF pulses prolong the TR, thereby making the off-resonance induced artifacts more prominent. In addition, simulations show that the myocardial-to-blood signal contrast in b-SSFP sequences is significantly lower when the excitation flip angle is deliberately set to a value less than 46°, as compared to a higher flip angle, either to reduce SAR or due to RF inhomogeneity. In this regard, Sung and colleagues [4] have shown that significant B_1_ inhomogeneity exists even across a small region spanning the extent of the heart, and our results confirm their findings.

When the subject-specific local RF shimming is performed by using 2 RF transmit sources, the scalar metric (C_v_) used for describing RF inhomogeneity decreases by 42%. Additionally, the number of voxels that fall within ± 10% of the mean flip angle increases significantly.

Our results show that with RF shimming, cardiac cine b-SSFP images show significantly fewer changes in the myocardial signal intensity across left ventricular regions than without RF shimming. The cine images acquired both with and without RF shimming were corrected to account for the spatial variation of receive-coil sensitivity, using the coil sensitivity maps acquired as a part of the parallel imaging acquisition. Therefore, our results suggest, at the very least, that a significant portion of the signal variation across the myocardium in b-SSFP sequences at 3T is due to transmit B_1_ inhomogeneity, which is substantially reduced with local RF shimming.A couple of points are worth noting with respect to this analysis. First, the signal intensity variation across the segments of the left ventricular myocardium did not show any particular pattern, suggesting that B_1_ field is subject-specific. Secondly, by calculating the IU values, we were able to determine the extent of myocardial signal intensity variation within a subject and across subjects. A higher value of IU variation in bSSFP images obtained without local RF shimming, when compared to those obtained with local RF shimming, points to the deleterious effect of B_1_ field inhomogeneity and the need for RF field shimming. Furthermore, the variation in the B_1_ field is subject-specific, as the fat, muscle, blood, and bone compartments of each person are different. The B_1_ field distribution depends on the tissues’ electrical parameters, such as electric conductivity and permittivity, as well as the coupling of the RF field to the subject being imaged. Previous studies have shown that the B_1_ field distribution within the body can be approximated with simple tissue models. However, it is challenging to predict the B_1_ field in the clinical setting, because it is difficult to model the fat/muscle distribution for any given patient [10,20]. The results of our study show that there is no clear relationship between local B_1_ field homogeneity and the subject body type (measured by BSA, BMI, or the AP/RL ratio), either with or without local RF shimming. Likewise μ, C_v_, and the spread of the transmit amplitude and phase did not show any dependence on the BMI, BSA, and AP/RL ratio. These findings explain the inconsistent results obtained with the use of devices such as RF cushions. They also point to the need for subject-specific RF shimming. While, we did not perform an extensive study of the effect of the size of the shim volume, we did not find substantial changes in the mean flip angles or the Cv, as long as the RF shim volume was placed over the heart. We found this variation to be less than 2%, across the heart, even when the size of the shim box around the heart was changed by a factor of two along each encoding direction. Other pulse-sequence modifications, e.g., B_1_-insensitive RF pulses have been proposed for combating the detrimental effect of B_1_ inhomogeneity. When these B_1_-insensitive RF pulses are used as preparation pulses, e.g., a T_2_ preparation pulse for coronary artery imaging, their prolonged duration is not detrimental and can even be effective. However, the use of such pulses in sequences such as cine b-SSFP is more challenging for the reasons previously described. The ability to perform RF shimming by using multiple transmit sources provides additional flexibility in combating the deleterious effects of B_1_ inhomogeneity without increasing scan time.

Researchers have found that having a greater number of transmit RF channels can provide greater flexibility and control over B_1_field shimming [21-23]. Nevertheless, the potential benefit of more RF channels must be balanced against increased system complexity. Preliminary theoretical evaluations indicate that the incremental benefit of having more than 2 transmit RF channels is rather modest [11], but there have been few clinical evaluations of such systems to date, and these issues need to be evaluated further.

Although our study confirmed some overall conclusions of Mueller and colleagues [13], substantial differences exist between their study and ours, including differences in analytical techniques. We believe that by clarifying the explicit relationship of the quantitative metrics to patient body habitus, our study makes an important contribution. Moreover, by providing information regarding the relative power between the 2 transmit channels and the phase difference between the channels as a function of body habitus, our results enable readers to make an informed decision about whether a dual-transmit system is needed for cardiac imaging at 3T or whether it might be just as effective to use a predetermined combination of RF amplitude/phase settings for the 2 channels, which might provide “optimal” B_1_ shimming for a given anatomy across all patients. This information is not available elsewhere in the literature. Without it, the value of multi-transmit solutions will be influenced by commercial considerations.

Our study has some limitations. First is the relatively small number of subjects involved. Although the population was small, we included subjects with varying body types, resulting in a wide range of BSAs, BMIs, and AP/RL ratios. This wide range provided an idea of how the B_1_ field varies with body habitus, although a larger number of subjects would have added to the confidence of the study. In addition, we analyzed only the b-SSFP images clinically, although the difference between shimming and not shimming the local RF field could have been visually qualified in other commonly performed cardiac sequences such as BB-TSE, perfusion imaging, and delayed-enhanced viability imaging. As explained earlier in this section, we felt that b-SSFP images capture the cyclic constraint between TR and SAR, thereby severely limiting the other options (such as B_1_-insensitive RF pulses) available for RF field shimming. Also, the increased scan time limited our ability to perform these comparisons. Future studies that could add to our findings include investigations of B_1_ field homogeneity with other techniques, such as RF cushions and B_1_ field-insensitive RF pulses.

## Conclusion

In conclusion, with or without RF shimming, cardiac B_1_ field homogeneity does not depend on body type, as characterized by body mass index, body surface area, and anterior-posterior to right-left patient width ratio. Cardiac B_1_ field homogeneity can be significantly improved by performing local radiofrequency (RF) shimming with 2 independent RF transmit channels. The quality of balanced steady-state free-precession cine images is improved with RF shimming.

## Competing interests

1) Dr. Amol Pednekar is an employee of Philips Health Care, Houston, TX, USA.

2) Dr. Marc Kouwenhoven is an employee of Philips Healthcare, Best, The Netherlands.

## Authors’ contributions

*Study Design:* RK, AP, BC, RM. *Image Acquisition:* RK, AP, BC, RM. *Post-Processing of Data:* RK. *First Draft:* RK and RM. *Draft editing:* RK, AP, MK, BC, RM. All authors read and approved the final manuscript.
